# An open data repository and a data processing software toolset of an equivalent Nordic grid model matched to historical electricity market data

**DOI:** 10.1016/j.dib.2017.02.021

**Published:** 2017-02-13

**Authors:** Luigi Vanfretti, Svein H. Olsen, V.S. Narasimham Arava, Giuseppe Laera, Ali Bidadfar, Tin Rabuzin, Sigurd H. Jakobsen, Jan Lavenius, Maxime Baudette, Francisco J. Gómez-López

**Affiliations:** a SmarTS Lab, KTH Royal Institute of Technology, Stockholm, Sweden; b Statnett SF, Oslo, Norway; c Technical University of Denmark, Risø, Denmark; d Norwegian University of Science and Technology, Trondheim, Norway

**Keywords:** Electrical power systems, Electric power transmission, Smart grid, Power system modeling and simulation, Power system dynamics, Dynamic simulations, Power flow, Common Information Model (CIM), Modelica, Historical market data, Modeling, Simulation

## Abstract

This article presents an open data repository, the methodology to generate it and the associated data processing software developed to consolidate an hourly snapshot historical data set for the year 2015 to an equivalent Nordic power grid model (aka Nordic 44), the consolidation was achieved by matching the model׳s physical response w.r.t historical power flow records in the bidding regions of the Nordic grid that are available from the Nordic electricity market agent, Nord Pool.

The model is made available in the form of CIM v14, Modelica and PSS/E (Siemens PTI) files. The Nordic 44 model in Modelica and PSS/E were first presented in the paper titled “iTesla Power Systems Library (iPSL): A Modelica library for phasor time-domain simulations” (Vanfretti et al., 2016) [1] for a single snapshot. In the digital repository being made available with the submission of this paper (SmarTSLab_Nordic44 Repository at Github, 2016) [2], a total of 8760 snapshots (for the year 2015) that can be used to initialize and execute dynamic simulations using tools compatible with CIM v14, the Modelica language and the proprietary PSS/E tool are provided. The Python scripts to generate the snapshots (processed data) are also available with all the data in the GitHub repository (SmarTSLab_Nordic44 Repository at Github, 2016) [2].

This Nordic 44 equivalent model was also used in iTesla project (iTesla) [3] to carry out simulations within a dynamic security assessment toolset (iTesla, 2016) [4], and has been further enhanced during the ITEA3 OpenCPS project (iTEA3) [5]. The raw, processed data and output models utilized within the iTesla platform (iTesla, 2016) [4] are also available in the repository. The CIM and Modelica snapshots of the “Nordic 44” model for the year 2015 are available in a Zenodo repository.

## Graphical abstract

**Figure:**
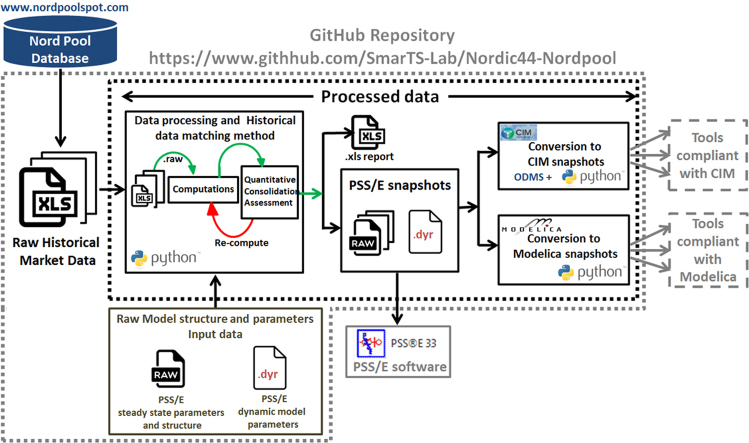

**Specifications Table**

**Table:** 

Subject area	*Electric Power Systems*
More specific subject area	*Power system dynamics and simulation*
Type of data	*Excel files, Tables, Figures*
How data was acquired	*Historical Data Records: Nord Pool;*
	*Source Model Parameter Data: Literature*[6], [7]
Data format	*Raw, Processed*
Experimental factors	*Power flows were obtained for the Nordic 44 model in PSS/E for every one hour for the year 2015 and were consolidated by matching the model׳s physical response with data records available from Nord Pool.*
Experimental features	*CIM v14 files and Modelica records were generated from the consolidated/matched PSS/E snapshots of the Nordic 44 model.*
Data source location	*Electric market data for the Nordic grid*
	*Nord Pool webpage (*http://www.nordpoolspot.com*) (for historical power flow data), model structure data from*[6], [7]*and modifications documented in this article.*
Data accessibility	*The processed data is archived in a Zenodo repository at:*
	*-*https://doi.org/10.5281/zenodo.162907 (2015 data)
	*The data processing software is made available in a GitHub repository:*
	Nordic44-Nordpool github.com/SmarTS-Lab/Nordic44-Nordpool

**Value of the data**

• The raw Nord Pool data of the power flow records matched with the model׳s response (processed data) yield thousands of representations of the Nordic 44 model that can be useful to understand the power flow patterns and the electricity market׳s operation in the Nordic synchronous electric power system during 2015.
• The Nordic 44 model can be used as a test system for power system studies, including static and dynamic analysis under realistic operation conditions for 2015. For example, it can be used to train and test Machine Learning techniques (e.g. Decision Trees) and other computational techniques that are essential in the work flows used for dynamic security assessment of electrical power systems.
• The processed data and models can be used to test and validate the functionalities of power system security assessment software both offline and online dynamic assessment tools, e.g. iTesla [3,4], DSAT [8], SIGAURD [9].
• The data provided using the CIM v14 standard can be used to test the functional layer of applications in Smart Grid Architectures and most importantly, to quantitatively assess the interoperability of power system simulation tools that adopt CIM.
• The data and models provided in Modelica can be used with any Modelica compliant software tool to perform power system dynamic simulations and studies. When using OpenModelica [10], this provides researchers with a fully open source software environment for dynamic simulation.

## 1 Data

### 1.1 Matching historical market data

Raw data from Nord Pool was consolidated with a physical model representation of the Nordic grid by matching the measurement records to the model׳s power flow results (processed data). This has resulted in thousands of representations of the “Nordic 44” model for the year 2015 that are made available in the GitHub repository with the submission of this article.

These snapshots are provided in the form of CIM v14, Modelica and PSS/E (Siemens PTI) files. The Python scripts (i.e. software toolset) used to generate these snapshots (CIM v14, Modelica and PSS/E) are also made available in the repository.

### 1.2 Historical market data

The pre-processed (raw) data was downloaded from Nord Pool webpage. For each hour, the records contain the active power production and consumption data in the bidding regions of the Nordic grid and the active power exchange between them. These records were matched to an electrical grid model steady-state response, as explained next.

### 1.3 Model parameter and structure data

To match and consolidate the historical market data to a physical description of the power network, the Nordic 44 model was developed. Note that the aim here was to set a “base case”, from which multiple snapshots of actual measurements records could be mapped to the quantitative response from computations on a physical model of the grid that included both steady-state and dynamic analysis features.

The development of this source model/“data” consisted of (a) obtaining editable files in the PSS/E form to, (b) extend the model to represent better the Norwegian portions of the Nordic grid and to adapt it to modeling limitations in both the iTesla platform and the iPSL library (i.e. lack of HVDC link models).

*Step (a)* used source files which was stripped from user defined models and other equipment, and other additional modifications carried out by Emil Hillberg of STRI on behalf of Statnett SF. The resulting model of this step is archived in Models.zip in respective Zenodo repositories.
  Note that the starting model is an extension itself of the Nordic 23 bus model developed at SINTEF Energy Research in several steps [6]. The Nordic 23 bus model was developed from a 15 bus Nordic power system model developed at NTNU and the details of this model are explained in [7].
*Step (b)* included the assignment of bus bar names, grouping of busses according to the actual bidding region, and numerous other changes as described in the documentation available in./…/ SmarTSLab_Nordic44/00_Documentation/N44_changes.docx.

The model developed in Step (b) is used throughout the historical data matching and consolidation process to create snapshots of the actual operational conditions of the Nordic Grid for 2015. This base case can be found in the repository in the GitHub repository at./nordic44/models/ and archived in Models.zip in respective Zenodo repositories.

## 2 Experimental design, materials and methods

### 2.1 Pre-processed data

**Fig. 1:**
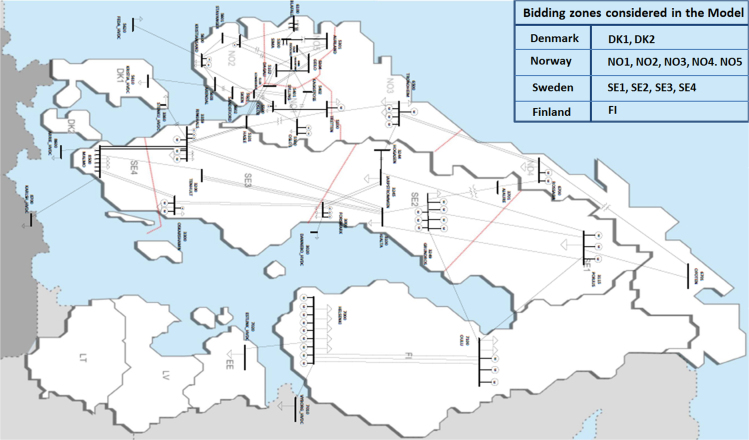
SmarTS Lab Nordic 44 Equivalent Model Mapped to the bidding zones of the Nordic grid used in Nord Pool for 2015.

The pre-processed (raw) data was downloaded from Nord Pool webpage as MS-Excel files (Consumption_xx.xlsx, Exchange_xx.xlsx, and Production_xx.xlsx) were saved in a folder with corresponding date (e.g. N44_20150401 refers to the folder containing the hourly snapshots of the 1^st^ April 2015). The MS-Excel files (Production_xx.xlsx and Consumption_xx.xlsx) contain the active power production and consumption data in the bidding regions of the Nordic grid for every hour as shown in Fig. 1. The MS-Excel file (Exchange_xx.xlsx) contains the active power exchange data between the bidding regions of the Nordic grid for every hour. Now, the toolset was updated to automatically download the data from the Nord Pool ftp server, inspired by the work in [11]. The raw data is stored in a Python dictionary allowing for easy data manipulation in Python and integration with PSS/e through psspy. It is still possible to save the data to excel and to load in excel files to the dictionary ensuring both forward and backward compatibility.

### 2.2 Data processing method

**Fig. 2:**
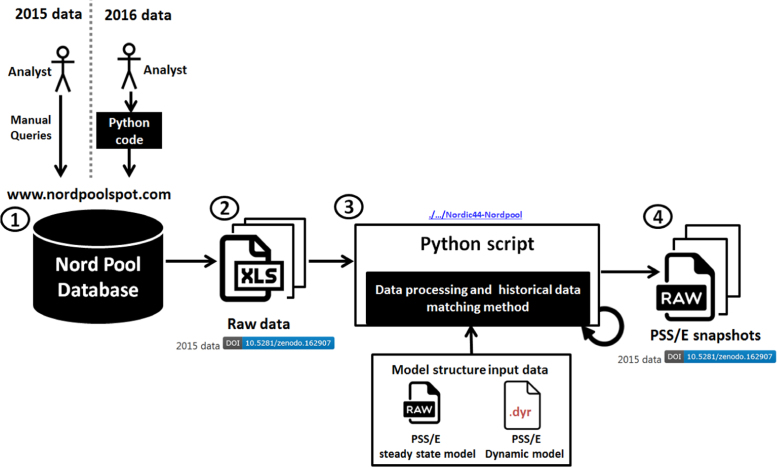
Matching and consolidation of historical data with the Nordic 44 grid model.

For each hour, an equivalent Nordic power system model (Nordic 44) was created, matched and consolidated with the load generation balance in the bidding regions and the active power exchange between the bidding regions. The buses in the Nordic 44 model were named according to the closest city or town corresponding to both the geographical location and the detailed grid structure available to Statnett SF. The work flow used to create the PSS/E snapshots is shown below in Fig. 2.

The major steps of the data processing workflow include:

(1) The raw data for the year 2015 was downloaded from Nord Pool (http://www.nordpoolspot.com) to MS-Excel files through manual queries to the Nord Pool database. This procedure was automated by implementing a Python class for connecting to the Nord Pool ftp server and handling the Nord Pool data. An example on how to use the Python code to generate the cases is provided in the GitHub repository (./examples/multiple_data_sets_from_nordpool.py /data_set_from_nordpool.py)
(2) The raw data from Nord Pool contains only active power flow measurements (generation, consumption and exchange) within the bidding regions and between the bidding regions, for every hour.
(3) Another Python script simulates/computes the power flow with the constraint of minimizing the error between the power through the lines between the bidding regions. The method implemented in the Python script performs several checks (e.g. convergence, limits etc.), and after completing these tasks, it computes the error between the Nord Pool measurement records and those obtained from the Python script computations on the Nordic 44 model. A summary of results is written in an MS-Excel file for each snapshot and named PSSE_in_out.xlsx.
(4) The obtained PSS/E snapshots (processed data) contain the power flow solutions that give the best match to the historical data from Nord Pool. These are necessary to initialize simulations, especially those needed for DSA.

**Fig. 3:**
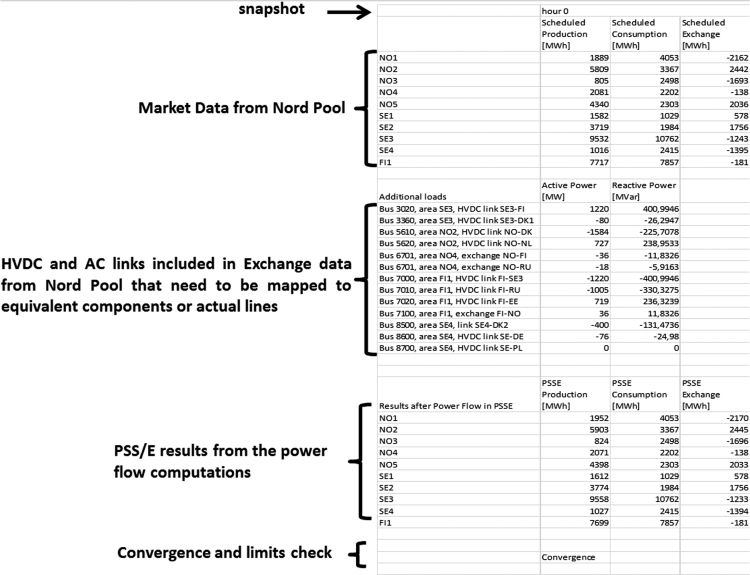
Screenshot of the MS-Excel file PSSE_in_out.xlsx.

At the end of this work flow, MS-Excel files (PSSE_in_out.xlsx) are generated by the Python script for every snapshot with raw data from Nord Pool and the results from PSS/E. These MS-Excel files (PSSE_in_out.xlsx) include limit checking messages (branch overloading, bus voltage out of limits and generator overloading). A screenshot of the created Excel file is shown in Fig. 3.

**Fig. 4:**
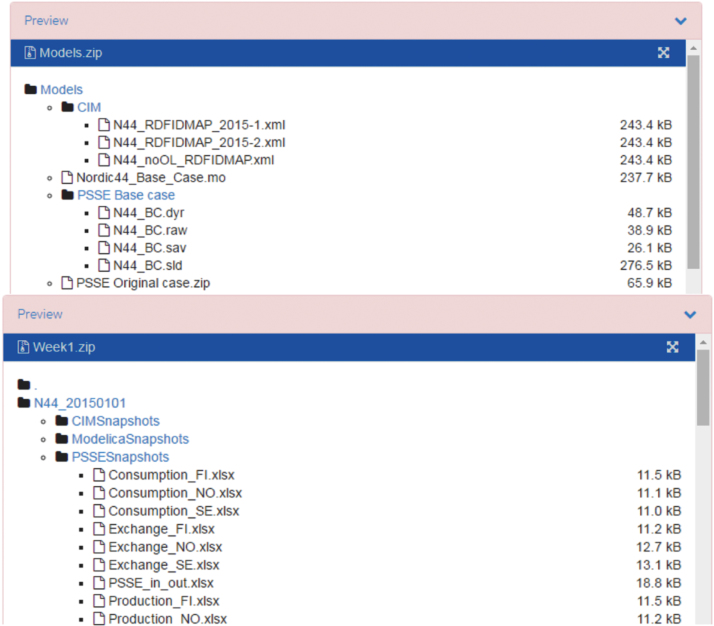
Screenshot of the Zenodo repository showing the Nordic 44 model and PSS/E snapshots.

The PSS/E snapshots for each hour before solving the power flow (hx_before_PF.raw, unmatched processed data) and after solving the power flow (hx_after_PF.raw, matched processed data) were also made available in the repository. The Nordic 44 model and the PSS/E snapshots can be accessed from the repository as shown in Fig. 4.

### 2.3 Processed data and post-processing

**Fig. 5:**
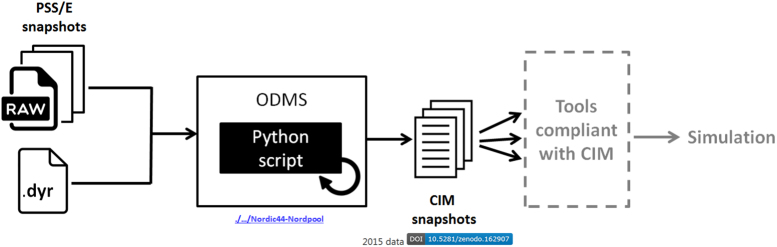
Generation of CIM v14 snapshots.

CIM v14 and Modelica snapshots were generated from the matched PSS/E solved power flow snapshots (processed data). The PSS/E snapshots and PSS/E dynamic model parameters data (.dyr) files were used by the Python script to generate the CIM v14 snapshots as shown in Fig. 5. The Python script uses the Application Programming Interface (API) of the Operational Database Management System (PSS/ODMS) software [12] to generate the snapshots. These generated CIM snapshots can be used for information exchange according to CIM and to perform analysis in CIM compliant tools [13], [14].

**Fig. 6:**
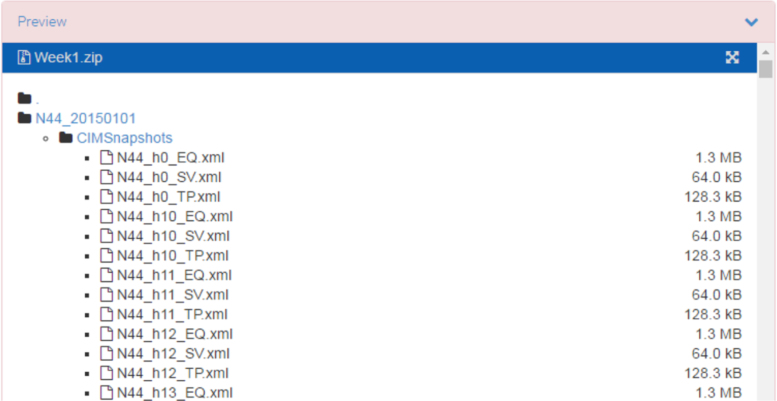
Screenshot showing the CIM v14 snapshots.

The generated CIM snapshots were placed in the folder corresponding to the day they refer to (e.g. N44_20150401 refers to the 1^st^ of April 2015). In each folder there are three files that define individual CIM snapshots for each hour (N44_hx_EQ.xml, N44_hx_SV.xml and N44_hx_TP.xml). N44_noOL_RDFIDMAP.xml is the file with IDs mapping of those cases with fixed overloading problems. N44_RDFIDMAP_2015-1.xml and N44_RDFIDMAP_2015-2.xml are the files with IDs mapping of the remaining snapshots from 2015. The screenshot of the generated CIM snapshots in the GitHub repository is shown in Fig. 6.

**Fig. 7:**
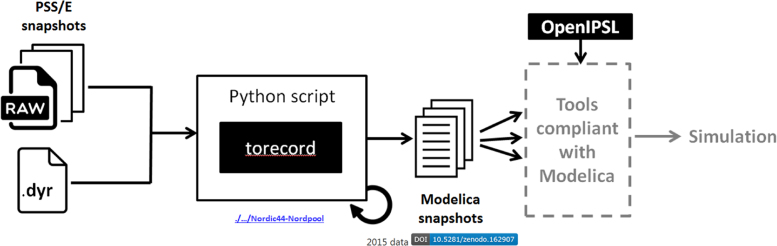
Generating Modelica snapshots.

PSS/E snapshots and PSS/E dynamic model parameters data (.dyr) files were used by the Python script “*Raw2Record”* (./…/SmarTS-Lab/Raw2Record) to generate the associated Modelica snapshots as shown in Fig. 7. These generated Modelica snapshots together with the OpenIPSL library can be used for simulation in the Modelica compliant tools. The generation of the record files using the python script is illustrated with an example (./examples/multiple_data_sets_from_nordpool.py /data_set_from_nordpool.py) and is provided in the GitHub repository.

During the iTesla project, another methodology to generate Modelica model snapshots using the iTesla platform [3] was attempted. Note that these snapshots were created using a different methodology reported in [15]. The resulting snapshots available for this approach cover only from April 1, 2015 to July 31, 2015 and are archived in iTesla_Platform.zip in the Zenodo repository. This archive contains both the snapshot models together with the appropriate version of the iPSL library.

**Fig. 8:**
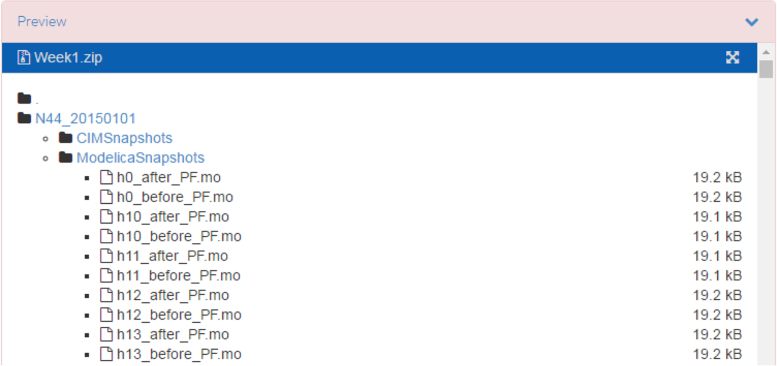
Screenshot to access Modelica snapshots.

The data records are stored in the /ModelicaSnapshots/ sub-folder of the Zenodo archive. They require the Modelica model that was manually implemented for Nordic 44 reported in [1] with the record structures corresponding to the PSS/E snapshots. The Python script (“torecord”) used to generate Modelica snapshots from PSS/E snapshots as described in Fig. 7. The OpenIPSL library used in generation of these snapshots can be found in./…/SmarTS-Lab/OpenIPSL (Fig. 8).

The CIM and Modelica snapshots (processed data) of the “Nordic 44” model for the year 2015 are available the aforementioned Zenodo repository (see [2]).
