# Supplementary material for: An open data repository and a data processing software toolset of an equivalent Nordic grid model matched to historical electricity market data
Source: Data Brief. 2017 Feb 13;11:349–57. doi: 10.1016/j.dib.2017.02.021 (PMC5328940; doi:10.1016/j.dib.2017.02.021)
Supplement: Supplementary file 1 — Supplementary material [file mmc1.pdf]

An Open Data Repository and a Data Processing Software Toolset of an Equivalent Nordic Grid Model Matched to Historical Electricity Market Data

Manuscript No.: DIB-D-16-00846

Authors' Declaration

We wish to confirm that there are no known conflicts of interest associated with this publication and there has been no significant financial support for this work that could have influenced its outcome.
We confirm that the manuscript has been read and approved by all named authors and that there are no other persons who satisfied the criteria for authorship but are not listed. We further confirm that the order of authors listed in the manuscript has been approved by all of us.
We confirm that we have given due consideration to the protection of intellectual property associated with this work and that there are no impediments to publication, including the timing of publication, with respect to intellectual property. In so doing we confirm that we have followed the regulations of our institutions concerning intellectual property.
We understand that the Corresponding Author is the sole contact for the Editorial process (including Editorial Manager and direct communications with the office). He/she is responsible for communicating with the other authors about progress, submissions of revisions and final approval of proofs. We confirm that we have provided a current, correct email address which is accessible by the Corresponding Author and which has been configured to accept email from: luigiv@kth.se, and luigi.vanfretti@gmail.com

Signed by all authors as follows: (attached pages with the corresponding signatures of some of the authors' complement this page, as some of the authors are not located in the same institution).

L. Vanfretti,	Date: 2016-12-14,	Signature:
S.H. Olsen,	Date: 2016-12-____,	Signature:
V.S. Narasimham Arava	Date: 2016-12-12,	Signature:
G. Laera	Date: 2016-12-13,	Signature: G. Laera
A. Bidadfar	Date: 2016-12-____,	Signature:
T. Rabuzin	Date: 2016-12-12,	Signature:
S.H. Jakobsen	Date: 2016-12-12,	Signature:
J. Lavenius	Date: 2016-12-12,	Signature:
M. Baudette	Date: 2016-12-12,	Signature:
F.J. Gómez Lopez	Date: 2016-12-12,	Signature:

An Open Data Repository and a Data Processing Software Toolset of an Equivalent Nordic Grid Model Matched to Historical Electricity Market Data

Manuscript No.: DIB-D-16-00846

Authors' Declaration

We wish to confirm that there are no known conflicts of interest associated with this publication and there has been no significant financial support for this work that could have influenced its outcome.
We confirm that the manuscript has been read and approved by all named authors and that there are no other persons who satisfied the criteria for authorship but are not listed. We further confirm that the order of authors listed in the manuscript has been approved by all of us.
We confirm that we have given due consideration to the protection of intellectual property associated with this work and that there are no impediments to publication, including the timing of publication, with respect to intellectual property. In so doing we confirm that we have followed the regulations of our institutions concerning intellectual property.
We understand that the Corresponding Author is the sole contact for the Editorial process (including Editorial Manager and direct communications with the office). He/she is responsible for communicating with the other authors about progress, submissions of revisions and final approval of proofs. We confirm that we have provided a current, correct email address which is accessible by the Corresponding Author and which has been configured to accept email from: luigiv@kth.se, and luigi.vanfretti@gmail.com

Signed by all authors as follows: (attached pages with the corresponding signatures of some of the authors' complement this page, as some of the authors are not located in the same institution).

L. Vanfretti,	Date:	2016-12-____,	Signature:	____________
S.H. Olsen,	Date:	2016-12-11,	Signature:	[signature]
V.S. Narasimham Arava	Date:	2016-12-____,	Signature:	____________
G. Laera	Date:	2016-12-____,	Signature:	____________
A. Bidadfar	Date:	2016-12-____,	Signature:	____________
T. Rabuzin	Date:	2016-12-____,	Signature:	____________
S.H. Jakobsen	Date:	2016-12-____,	Signature:	____________
J. Lavenius	Date:	2016-12-____,	Signature:	____________
M. Baudette	Date:	2016-12-____,	Signature:	____________
F.J. Gómez-López	Date:	2016-12-____,	Signature:	____________

An Open Data Repository and a Data Processing Software Toolset of an Equivalent Nordic Grid Model Matched to Historical Electricity Market Data

Manuscript No.: DIB-D-16-00846

Authors' Declaration

We wish to confirm that there are no known conflicts of interest associated with this publication and there has been no significant financial support for this work that could have influenced its outcome.
We confirm that the manuscript has been read and approved by all named authors and that there are no other persons who satisfied the criteria for authorship but are not listed. We further confirm that the order of authors listed in the manuscript has been approved by all of us.
We confirm that we have given due consideration to the protection of intellectual property associated with this work and that there are no impediments to publication, including the timing of publication, with respect to intellectual property. In so doing we confirm that we have followed the regulations of our institutions concerning intellectual property.
We understand that the Corresponding Author is the sole contact for the Editorial process (including Editorial Manager and direct communications with the office). He/she is responsible for communicating with the other authors about progress, submissions of revisions and final approval of proofs. We confirm that we have provided a current, correct email address which is accessible by the Corresponding Author and which has been configured to accept email from: luigiv@kth.se, and luigi.vanfretti@gmail.com

Signed by all authors as follows: (attached pages with the corresponding signatures of some of the authors' complement this page, as some of the authors are not located in the same institution).

L. Vanfretti,	Date: 2016-12-____,	Signature: ____________
S.H. Olsen,	Date: 2016-12-_____,	Signature: ____________
V.S. Narasimham Arava	Date: 2016-12-_____,	Signature: ____________
G. Laera	Date: 2016-12-_____,	Signature: ____________
A. Bidadfar	Date: 2016-12-12,	Signature: ____________
T. Rabuzin	Date: 2016-12-_____,	Signature: ____________
S.H. Jakobsen	Date: 2016-12-_____,	Signature: ____________
J. Lavenius	Date: 2016-12-_____,	Signature: ____________
M. Baudette	Date: 2016-12-_____,	Signature: ____________
